# Computational Analysis Predicts Correlations among Amino Acids in SARS-CoV-2 Proteomes

**DOI:** 10.3390/biomedicines11020512

**Published:** 2023-02-10

**Authors:** Emmanuel Broni, Whelton A. Miller

**Affiliations:** 1 Department of Medicine, Loyola University Medical Center, Loyola University Chicago, Maywood, IL 60153, USA; 2 Department of Molecular Pharmacology & Neuroscience, Loyola University Medical Center, Loyola University Chicago, Maywood, IL 60153, USA

**Keywords:** amino acid composition, amino acid prediction, SARS-CoV-2, proteome, mutation, frequency, z-score

## Abstract

Severe acute respiratory syndrome coronavirus 2 (SARS-CoV-2) is a serious global challenge requiring urgent and permanent therapeutic solutions. These solutions can only be engineered if the patterns and rate of mutations of the virus can be elucidated. Predicting mutations and the structure of proteins based on these mutations have become necessary for early drug and vaccine design purposes in anticipation of future viral mutations. The amino acid composition (AAC) of proteomes and individual viral proteins provide avenues for exploitation since AACs have been previously used to predict structure, shape and evolutionary rates. Herein, the frequency of amino acid residues found in 1637 complete proteomes belonging to 11 SARS-CoV-2 variants/lineages were analyzed. Leucine is the most abundant amino acid residue in the SARS-CoV-2 with an average AAC of 9.658% while tryptophan had the least abundance of 1.11%. The AAC and ranking of lysine and glycine varied in the proteome. For some variants, glycine had higher frequency and AAC than lysine and vice versa in other variants. Tryptophan was also observed to be the most intolerant to mutation in the various proteomes for the variants used. A correlogram revealed a very strong correlation of 0.999992 between B.1.525 (Eta) and B.1.526 (Iota) variants. Furthermore, isoleucine and threonine were observed to have a very strong negative correlation of −0.912, while cysteine and isoleucine had a very strong positive correlation of 0.835 at *p* < 0.001. Shapiro-Wilk normality test revealed that AAC values for all the amino acid residues except methionine showed no evidence of non-normality at *p* < 0.05. Thus, AACs of SARS-CoV-2 variants can be predicted using probability and z-scores. AACs may be beneficial in classifying viral strains, predicting viral disease types, members of protein families, protein interactions and for diagnostic purposes. They may also be used as a feature along with other crucial factors in machine-learning based algorithms to predict viral mutations. These mutation-predicting algorithms may help in developing effective therapeutics and vaccines for SARS-CoV-2.

## 1. Introduction

Proteins play crucial roles in many biological processes in organisms and they remain a major source of druggable targets [1]. Protein homologs usually have similar structures and functions [2]. In addition, proteins belonging to the same family and having related shapes tend to interact with other molecules in similar ways. Proteins bind to other molecules in order to complete specific tasks, which is highly influenced by the way the protein’s exposed surfaces interact with the molecules. The proteome of an organism consists of the proteins that are or can be expressed by a cell, tissue or organism, including intrinsically disordered proteins (IDP) or regions (IDR) [3,4].

Proteins are formed by long chains of amino acids which are connected via covalent peptide bonds [5,6]. Recent studies have analyzed the amino acids that make up proteins and have linked the sequence and composition to the protein’s function and shape. A protein’s amino acid composition (AAC) can be defined as the percentages of each amino acid in the sequence of that protein [7,8]. The AAC and sequence of amino acid residues are responsible for the native structure and functionality of a protein in an environment. Various factors can influence the AACs of proteins in organisms. The guanine-cytosine (G-C) content is the most important determinant of AAC [9,10]. The habitat of an organism also plays a key role in its AAC [11,12,13]. This is evident in adaptation of organisms to different habitats and landscapes [13]. Previous studies have proposed the use of relative AAC as a signature of a habitat or environment although the AAC is not highly determined by the environment as compared to the G-C content of the organism [13].

Various studies have successfully developed models that predict the structural class of proteins by using the AAC [14,15] since there is a strong correlation between AAC and structural class of a protein [16,17]. Sufficient knowledge about the structural class of proteins is a major boost for protein structure prediction, which in turn has significant medical and pharmaceutical implications [18]. Other studies have also suggested exploiting AAC for predicting protein-protein interactions [19,20]. Protein secondary structure can also be predicted using the protein’s AAC along with evolutionary information [21].

The severe acute respiratory syndrome coronavirus 2 (SARS-CoV-2) has caused a global health crisis and seems to be unending. The rate of mutation of the virus is increasing the challenges of finding suitable therapeutic options [22,23,24,25]. Virus mutation is a natural process that occurs when the virus replicates. Errors can occur in different parts of the virus’s genome during replication, resulting in changes to the genetic code of the virus. Some mutations can result in changes to the virus’s antigenicity [26,27,28], making it more or less recognizable to the immune system, while others can affect its transmissibility [29,30,31,32], virulence, or ability to evade host immune response [26,27,28]. Mutations can arise spontaneously [33,34,35] or through selective pressure including exposure to antiviral drugs (antiviral resistance) [36,37] or even host immune responses (antigenic drift) [38,39,40]. Some mutations can have a significant impact on the virus’s biology and behavior, while others have little or no effect. The RNA virus, SARS-CoV-2, uses RNA as its genetic material and RNA replication has been shown to be less accurate than DNA replication [41]. The low fidelity of the RNA-dependent RNA polymerase used by SARS-CoV-2 to replicate its genetic material [42] leads to replication errors and higher mutational loads responsible for the emergence of variants [43]. Most RNA viruses lack a proofreading domain thus the increased frequency of mutations [44,45,46,47]. However, the SARS-CoV-2 has an exoribonuclease (ExoN) found on the non-structural protein 14 (nsp14) which it uses for proofreading although it still has its limitations [45,48,49,50], evident in the very high mutation rate of the virus [51,52].

RNA recombination is another molecular mechanism the SARS-CoV-2 virus could use for mutation, as a high frequency of recombination events occur in coronaviruses [53]. A case of intra-host SARS-CoV-2 recombination during a coinfection by the Delta (AY.33) and Gamma (P.1) has been reported [54]. A total of six recombinant regions across the genome within the same sample were identified: four in the spike gene and two in the nucleocapsid gene [54]. Computational analysis also predicted the possibility of future recombination events between SARS-CoV-2 and the Middle East respiratory syndrome coronavirus (MERS-CoV) RNA [55]. The accumulation of multiple mutations over time can result in the emergence of new viral variants. Understanding these molecular mechanisms, pattern and rate at which the virus mutates will be a boost to producing therapeutic molecules that will target future variants.

The living world is full of patterns which may not be easily identified [56,57]. These patterns may follow mathematical principles such as the Hardy-Weinberg Law [58], Benford’s Law [59,60], among others. Proteins also have patterns in various regards, and these patterns may be guided by mathematical or statistical functions and principles [61]. These patterns could be greatly influenced by the relationships shared among the amino acids that make up the protein. Herein, we sought to investigate the relationship that exists among amino acid residues in the various SARS-CoV-2 proteomes. The correlations may provide insights into the mutual constrained conditions of amino acids in the various SARS-CoV-2 variants during evolution.

## 2. Materials and Methods

### 2.1. Retrieving Amino Acid Sequences

FASTA files containing the amino acid sequences of the various SARS-CoV-2 proteins were retrieved from the National Center for Biotechnology Information (NCBI) database (https://www.ncbi.nlm.nih.gov accessed on 10 September 2022) using the keywords SARS-CoV-2, complete genome and the “Pango lineage” [62]. The sequences were retrieved using the Pango lineage nomenclature classification of the various SARS-CoV-2 variants [63] which were obtained from CoVariants (https://www.covariants.org/variants accessed on 10 September 2022) and the World Health Organization (WHO) Tracking SARS-CoV-2 variants (https://www.who.int/activities/tracking-SARS-CoV-2-variants accessed on 10 September 2022).

### 2.2. Data Pre-Processing

Based on Pango lineage, the amino acid sequences were split into separate files. All lines in each text or FASTA file that do not contain amino acid residues were removed using the “sed” command on linux terminal. Each file was then stripped of newlines and whitespace characters in order to combine all the sequences as one continuous string. The files were then read and saved into “DataFrames” based on their lineages using Pandas, an open source data analysis and manipulation tool for Python [64,65]. All strains that had non-standard amino acid residues in their proteome sequences were removed from the data.

### 2.3. Analysis

#### 2.3.1. Determining Amino Acid Frequencies

For each strain (or sample), the frequencies and AACs of the standard amino acids were determined using the “counter” function in Python and saved into “Dataframes”. The average AACs per variant were also determined by calculating the percentages of each residue per variant and for the virus as a whole. Furthermore, the standard deviations were computed and stored. The “Dataframes” were then exported as Excel workbooks.

#### 2.3.2. Correlation Analyses

Correlation analyses were performed on the SARS-CoV-2 variants and the amino acid residues to investigate their relationships based on their AACs. R studio was employed to generate the correlation plots for the amino acid residues after loading the Excel workbooks. The “Hmisc” package in R was used to generate the correlation coefficients with their significance levels (*p*-values) by using the “rcorr” function. The “corrplot” package was then used to plot the correlograms.

#### 2.3.3. Test for Normality

The study further tested the normality of each amino acid composition’s distribution using the Shapiro-Wilk test, skewness, and kurtosis. Using the “shapiro” function in the “scipy” library, the test statistic and corresponding *p*-values were determined. The “skewtest” and “kurtosis” functions were employed to compute the skewness and kurtosis of the distribution, respectively. P-P plots were also generated to visualize the normality of the amino acid AAC distributions.

### 2.4. Validating Correlation Analysis

A multiple template alignment tool, WebPRANK was employed to align a maximum of 3 proteome sequences from each variant/lineage [66]. For variants that had fewer than 3 proteome files, all the available sequences were used. In all, a total of 28 sequences were aligned: three each from B.1.1.7, B.1.351, P.1, B.1.617.2, B.1.525, B.1.526, C.37, and BA.1, two from BA.4, and one each from B.1.177 and B.1.160. The samples are labelled as “A”, “B” and “C” to enable readability of the analysis. The output from the alignment was then compared to the AAC analyses.

## 3. Results and Discussion

Based on the search via the National Center for Biotechnology Information (NCBI) database, the data obtained were grouped into 12 variants (Table 1) comprising of:

1. B.1.1.7 (Alpha)
2. B.1.351 (Beta)
3. P.1 (Gamma)
4. B.1.617.2 (Delta) and B.1.617.1 (Kappa)
5. B.1.525 (Eta)
6. B.1.526 (Iota)
7. C.37 (Lambda)
8. BA.1 and BA.2 (both Omicron)
9. BA.4 (Omicron)
10. BA.5 (Omicron)
11. B.1.177
12. B.1.160.

All proteome sequences that had non-standard or ambiguous amino acid residues such as Xaa (unspecified or unknown amino acid, X), Glx (glutamine or glutamic acid, Z), Asx (asparagine or aspartic acid, B), and Xle (leucine or isoleucine, J) were removed from the dataset. All 23 proteome sequences of BA.5 had one or more of the non-standard amino acid residues thus, were not included in this study (Table 1). For B.1.1.7 (Alpha), 706 out of the 830 were used in this study. For the B.1.526 (Iota) variant, 773 out of the 3085 passed this criterion and were used in the study. Variants B.1.525, B.1.160, and B.1.177 did not have any of the ambiguous amino acids in any of the datasets. In all, a total of 1637 complete SARS-CoV-2 proteome sequences belonging to 11 variants/lineages were used for the analysis (Table 1).

### 3.1. The Range of Amino Acid Residues in the SARS-CoV-2 Samples

The number of amino acid residues in the SARS-CoV-2 proteomes used herein ranged from 13,925 to 14,153. Most B.1.1.7 samples were observed to either have a total amino acid residue count of 14,045 or 14,149 while all 4 samples of B.1.351 had a total residue count of 14,140. All the 13 B.1.525 samples had a total residue count of 14,138. For B.1.526, most of the samples had either 14,143 or 14,142 residues. Other B.1.526 samples had total amino acid residue counts of 14,045, 14,149, 14,138, 14,147, 14,147, 14,149 or 14,140. For B.1.617.2, two samples had a total residue count of 13,926, while most of the samples had either 14,149 or 14,145. Other B.1.617.2 samples had 14,026 or 14,139 and one sample had 14,140 residues. The BA.1 samples used herein had total residue counts ranging from 14,129 to 14,145, while the two samples of BA.4 had 14,129 and 14,135 residues. All eight C.37 samples had a total residue count of 14,136. Computing the complete amino acid count considering all 17 proteins in the SARS-CoV-2 proteome revealed a total of ~14,439 residues. The inconsistency in the total residue counts obtained herein may be due to deletions and insertions in various SARS-CoV-2 genome nucleotides, resulting in fewer or more amino acid residues in the proteins, respectively [67,68]. For instance, an 81 base-pair deletion was observed in a SARS-CoV-2 case in Arizona, resulting in a 27 amino acid deletion in the Open reading frame 7a (ORF7a) protein [67]. Several other deletions have been reported in the SARS-CoV-2 spike protein [38,69,70] and non-structural protein 1 (nsp1) [71,72,73]. Amino acid deletions in the other SARS-CoV-2 proteins have also been highlighted in literature [68,74]. In addition, some SARS-CoV-2 ORF proteins such as ORF3c (~41 amino acids) are translated during infection [75,76]. The sample data obtained from NCBI did not contain sequences of ORF9b (9b) [~97 amino acids], ORF9c (9c) [~73 amino acids], ORF3b (3b) [~22 amino acids], ORF3c (3c) [~41 amino acids], and ORF3d (3d) [~57 amino acids] thus, the lower number of amino acid residues. Without these 5 proteins, the amino acid count is ~14,149, consistent with those obtained herein.

### 3.2. Amino Acid Frequencies per Variant

**Figure 1:**
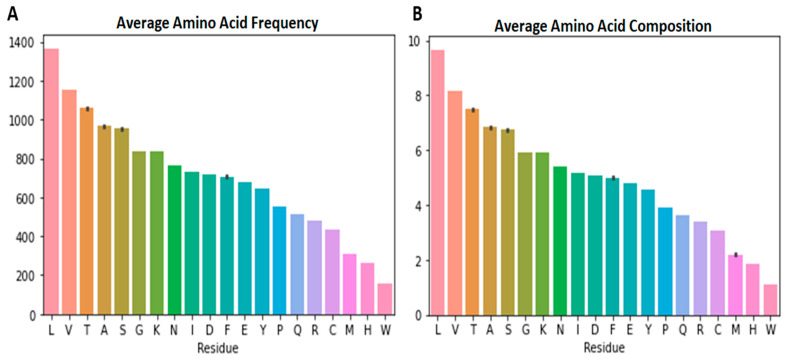
Average amino acid frequency and composition plots for SARS-CoV-2: (**A**) Average amino acid frequency and (**B**) average amino acid composition of all the SARS-CoV-2 proteomes.

The frequencies of the 20 standard amino acids in each of the 1637 complete SARS-CoV-2 proteomes were determined. For each of the 11 variants, the average frequencies of the amino acid residues were also determined (Table 2). The average amino acid frequencies of all SARS-CoV-2 variants along with their standard deviations were also computed using the mean frequencies of each variant (Figure 1A and Table S1). The variant averages were used in order to avoid biases owing to the uneven data sets of the variants (the uneven number of samples for each variant). It was observed that leucine had the highest mean frequency of 1365.131 (Table S1) in the SARS-CoV-2 proteomes, ranging from 1362 (BA.4) to 1370 (B.1.177) [Table 2]. Tryptophan was observed to have the least mean frequency of 156.895 (Table S1). Variants B.1.525, C.37, BA.1, BA.4, B.1.177, and B.1.160 were observed to have 157 tryptophans in their proteomes while B.1.1.7, B.1.351, P.1, B.1.617.2, and B.1.526 had, on average, 156.469, 156.75, 156.959, 156.721, and 156.951 tryptophans, respectively. On average, the B.1.617.2 variant was observed to have the most Met count of 312.512, while B.1.160 had the least (309) [Table 2].

The standard deviation provides a measure to determine the dispersion of the data from the mean [77]. The standard deviation of the amino acid frequency provides information on the extent of mutation by each residue in the various variants. On average, threonine was observed to have the highest standard deviation of 3.743 followed by isoleucine, glycine, proline, phenylalanine, lysine, and serine with 3.11, 2.467, 2.421, 2.357, 2.337, and 2.296, respectively (Table S1), signifying the relatively higher involvement of these residues in SARS-CoV-2 mutations. Tryptophan, cysteine and methionine on the other hand, were observed to have the least standard deviations of 0.175, 0.864, and 0.95, respectively (Table S1), implying that they are less involved in SARS-CoV-2 mutations.

### 3.3. Highest and Least Represented Amino Acids in the SARS-CoV-2 Proteomes

**Figure 2:**
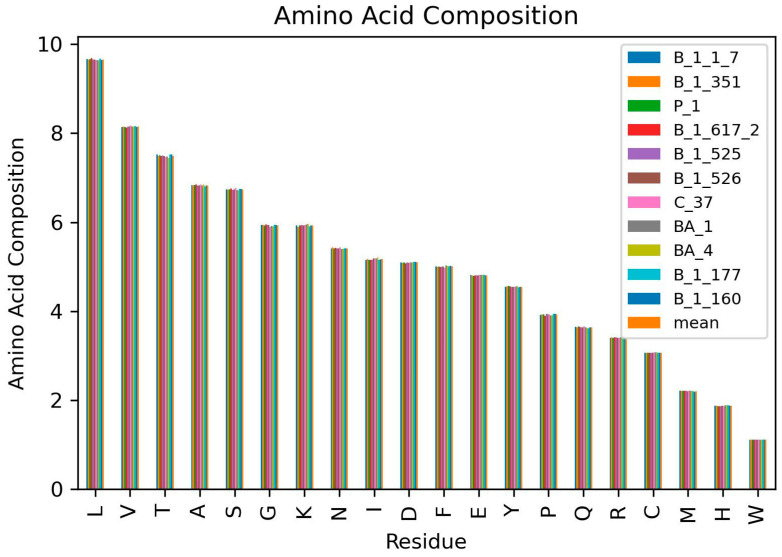
Amino acid composition of the 11 SARS-CoV-2 variants.

Due to the inconsistency in amino acid frequencies, the AAC for the various SARS-CoV-2 variants were determined (Figure 2 and Table 3). The AAC provides a more accurate metric to compare the amino acids in the various SARS-CoV-2 proteomes since it deals with the percentage of each residue in the proteome. The AAC caters for the inconsistencies in residue frequencies caused by insertion or deletion of genomic nucleotides. Proteome-wide analysis of the various SARS-CoV-2 variants showed that Leu was the most abundant amino acid with an average AAC of 9.658% while Trp was the least with an average AAC of 1.110% (Figure 1B and Table 3). Leucine is the most abundant amino acid in proteomes [78] and has been shown to be the most abundant in cyanobacterial proteomes [79] and even plant proteomes from 144 plant species [80]. Herein, valine, threonine, alanine and serine are the other highly abundant amino acids with average AACs of 8.149, 7.493, 6.831, and 6.739%, respectively (Figure 1B, Figure 2 and Table 3). In plant proteomes, Trp was also observed to be the least encoded [80]. Other low abundant SARS-CoV-2 amino acids in addition to Trp include His, Met, and Cys with average AAC values of 1.873, 2.205, and 3.070%, respectively (Table 3). Trp and Cys have previously been reported to be the least abundant in proteins found in Swissprot and TrEMBL databases [78]. A previous study showed that cysteine abundance positively correlates with the complexity of the organism although cysteine is underrepresented in all organisms [81]. Cysteine occurrence in *Thermus aquatcus*, *Haloarcula maresmortui*, *Escherichia coli*, *Saccharomyes cerevisiae*, *Drosophila*, and human proteins were 0.4, 0.5, 1.1, 1.3, 1.9, and 2.3%, respectively [81]. Although coronaviruses are structurally complex [82,83,84], the classification of viruses as organisms is highly debated. Investigating the cysteine occurrence in different viruses is necessary to determine if viral complexity (among viruses) also correlates with the cysteine content. On average, almost 55.61% of the SARS-CoV-2 proteome were observed to be non-polar amino acids while 44.39% were polar (Table 3).

The average AACs per variant were also determined in order to analyze the highest and lowest abundant amino acids for each variant. The AACs can help determine the relationship between variants since AACs are signatures for organisms and environments [13]. Variant B.1.617.2 had the most abundant Leu, Ala, Gly, and Met with average AACs of 9.688, 6.846, 5.951, and 2.215%, respectively. Variants B.1.617.2, C.37, and BA.4 had the highest Trp content in their proteomes with an AAC of 1.111%. SARS-CoV-2 variant C.37 was observed to have the most abundant Glu, Ser, Val, and Asn with AACs of 4.817, 6.768, 8.168, and 5.429%, respectively. Variant BA.4 was observed to have the highest abundant Lys (5.958%), His (1.889%), Cys (3.078%), Tyr (4.568%), Ile (5.215%), and Arg (3.418%). For residues Pro, Phe, and Gln, variants B.1.526, BA.1, and P.1 had the highest abundance with corresponding AACs of 3.94, 5.025, and 3.66% (Table 3). For Thr, variants B.1.177 and B.1.160 had the highest abundance with an AAC of 7.52%. Both B.1.177 and B.1.160 also had similar Ser, Pro, Arg, Asp, Cys, and Trp compositions of 6.75, 3.937, 3.4, 5.103, 3.067, and 1.11%, respectively. They both had the most abundance for Asp (5.103%). These could imply that both B.1.177 (EU1) and B.1.160 (EU2) share the same ancestry as confirmed by the phylogenetic relationships of Nextstrain SARS-CoV-2 clades, reporting that B.1.177 (EU1) is an offspring of EU2. The signature A222V mutation of the spike protein of B.1.177 (20E EU1) variant was identified in two genomes of B.1.160 (20A EU2) [85]. Moreover, both variants were first identified in Europe and there is no evidence of increased viral transmissibility between them [86].

### 3.4. Tryptophan Less Likely to Mutate (Intolerant to Mutation)

Comparing the averages of the various variants, it was observed that tryptophan maintained an average frequency of 156.895 (157) and an average AAC of 1.11% in the SARS-CoV-2 proteome. Variants B.1.1.7, B.1.351, P.1, B.1.617.2, and B.1.526 had tryptophan average frequencies of 156.469, 156.75, 156.959, 156.721, and 156.951, respectively, while the rest maintained the 157 average count in their proteomes (Table 2). Variants P.1, B.1.525, B.1.526, BA.1, B.1.177, and B.1.160 had ~1.110% of their proteomes to be tryptophan, B.1.617.2, C.37, and BA.4 had 1.111%, while B.1.1.7 and B.1.351 had 1.109% as tryptophan (Table 3). Tryptophan was observed to be relatively equal in all the SARS-CoV-2 variants analyzed herein. Previous studies have shown that tryptophan in different SARS-CoV-2 proteins are conserved and replacing them might make the proteins unstable [87,88]. This may be due to the fact that tryptophan is encoded by only one codon, UGG. The paucity of the UGG codon in the genome makes it difficult to allow for any mutations, which might result in highly unstable proteins in the proteome as a whole. A recent study has shown that mutating tryptophan in the neucleocapsid (N) protein makes the virus very unstable [87]. Tryptophan has been reported to play an important role in the structural stability of proteins [89,90].

### 3.5. Lysine and Glycine Counts and Ranking

Sorting the amino acid frequencies and AACs in descending order revealed that the positions of lysine and glycine varied in the proteomes of the SARS-CoV-2 variants. Glycine and lysine had mean values of 838.220 and 838.047 with standard deviations of 2.467 and 2.337, respectively, for all SARS-CoV-2 variants. For variants B.1.1.7, B.1.160, B.1.177, B.1.351, B.1.525, B.1.526, B.1.617.2, and P.1, glycine had higher counts (ranked 6th most abundant) than lysine (7th most abundant) and vice versa for variants BA.1, BA.4, and C.37. For variants BA.1, BA.4, and C.37, lysine was the 6th most abundant while glycine was the 7th. Variants BA.1 and BA.4 both belong to the omicron variant of the SARS-CoV-2 while C.37 is a lambda variant. Lambda has been reported to share a common ancestry with omicron in contrast with delta [91]. Omicron and lambda also share common mutations in the genomic regions of ORF1b, spike protein and ORF8 [92]. The difference in lysine and glycine counts in the various SARS-CoV-2 variants necessitates the investigation of their effects in viral transmission, replication, survival, and other mechanisms. Lysine mutation (K417N and E484K) in the spike protein has been shown to significantly influence Angiotensin-converting enzyme 2 (ACE2) binding [93,94], suggesting lysine’s critical role in viral entry. An in silico structure-based energy calculation suggests that mutations at Gly431, Gly648, and Gly35 may cause massive destabilizing effects on the full-length spike protein [95]. In addition, variants with the D614G mutation were observed to be more dominant and had higher transmissibility than those with Asp614 by enhancing ACE2 binding affinity [31,96].

### 3.6. Correlation Analysis

**Figure 3:**
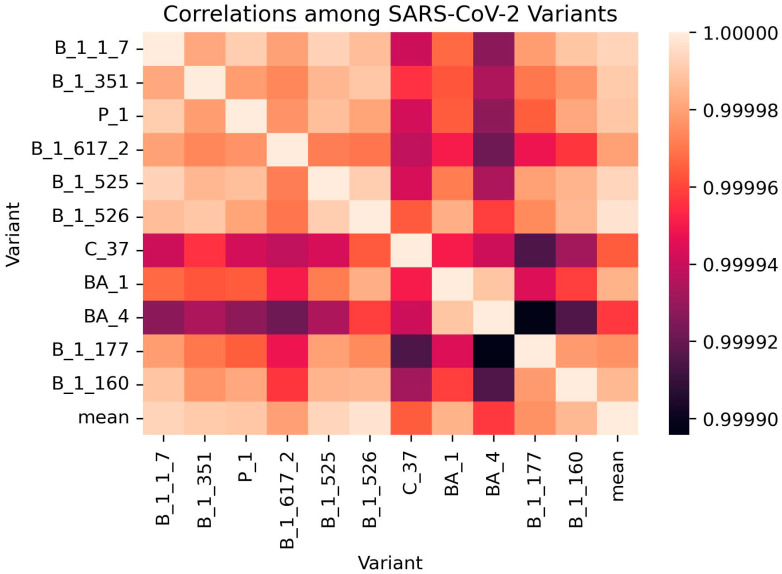
Correlation matrix of the various SARS-CoV-2 variants using their AACs.

A correlation matrix was generated for the SARS-CoV-2 variants using their AACs (Figure 3 and Table S2). The correlation coefficients among the variants ranged between 0.999895 and 1, supporting the fact that all the SARS-CoV-2 variants share a high degree of closeness (Table S2). The pair with the least correlation involved BA.4 and B.1.177 with a coefficient of 0.999896 while variants B.1.525 and B.1.526 had the highest correlation (0.999992) [Table S2]. B.1.525 (Eta) and B.1.526 (Iota) variants were both first discovered in New York in November 2020 and have been previously shown to have similar spike mutations including E484K and D614G [97]. B.1.351 (Beta, 20H) and B.1.526 (Iota, 21F) variants also demonstrated a very strong correlation of 0.99999, supporting existing studies that show that they both share a common ancestry (20C). Herein, the phylogenetic relationship of SARS-CoV-2 clades generated by Nextstrain [98] was used (available at https://github.com/nextstrain/ncov-clades-schema accessed on 12 December 2022) [Figure S2]. BA.4 [Omicron (22A)] and B.1.177 [20E (EU1)] were observed to have the least correlation of 0.999896. BA.4 was originally detected in South Africa [99,100] while B.1.177 was identified in Spain [86]. Furthermore, the phylogenetic tree by Nextstrain shows that BA.4 and B.1.177 are highly unrelated among the SARS-CoV-2 variants [Figure S2].

B.1.177 had relatively lower correlations with C.37 and BA.1 with coefficients of approximately 0.999915 and 0.999944, respectively (Table S2). Variants C.37, BA.1, and BA.4 were also observed to be the least correlated to the other variants (Figure 3). Variants B.1.1.7 and P.1 were observed to be highly correlated with a correlation coefficient of 0.999991. B.1.1.7 is the alpha variant [V1 (20I)], which shares a common ancestry [B.1.1 (20B)] with P.1 [gamma variant, V3 (20J)].

The B.1.526 variant was closest to the mean variant with the highest correlation coefficient of 0.999997 (Table S2). This result is consistent with the determined amino acid frequencies and compositions (Table 2 and Table 3). The AACs of B.1.526 and the mean were very similar while all other variants seem to deviate from the mean. For residues serine, proline and tryptophan, B.1.526 had the same AACs of 6.739, 3.934, and 1.110%, respectively, with the mean. Other residues including Met, Glu, Leu, Val, Phe, Asn, Lys, Arg, Asp, Ala, Cys and Tyr had very similar AACs for both B.1.526 and the mean. However, for proline and threonine the difference in AAC between B.1.526 and the mean were equal to or greater than 0.01%. For proline, B.1.526 and the mean had AACs of 3.934 and 3.924%, respectively, while 7.489 and 7.493%, respectively, were observed for Thr. B.1526 can be suitable as a reference for mutation studies, since it is closest to the mean and all the other variants deviate from the mean. Variants B.1.525, B.1.1.7, and B.1.351 also had very high correlation with the mean with coefficients of 0.999994, 0.999993, and 0.999991, respectively (Table S2). BA.4 was observed to be the least correlated to the mean with a coefficient of 0.999958 (Table S2).

C.37 had correlation coefficients of 0.999941 and 0.999942 with B.1.1.7 and P.1, respectively (Table S2). This is not surprising as C.37 has been shown to be an offspring of B.1.1.1, which in turn is an offspring of B.1.1. Alpha variant (B.1.1.7) and gamma variant (P.1) are also direct offspring of B.1.1. Surprisingly, the AAC of C.37 (lambda) had high correlation with those of B.1.526, B.1.351 and BA.1 with coefficients of 0.999964, 0.999956, and 0.99995, respectively (Table S2).

**Figure 4:**
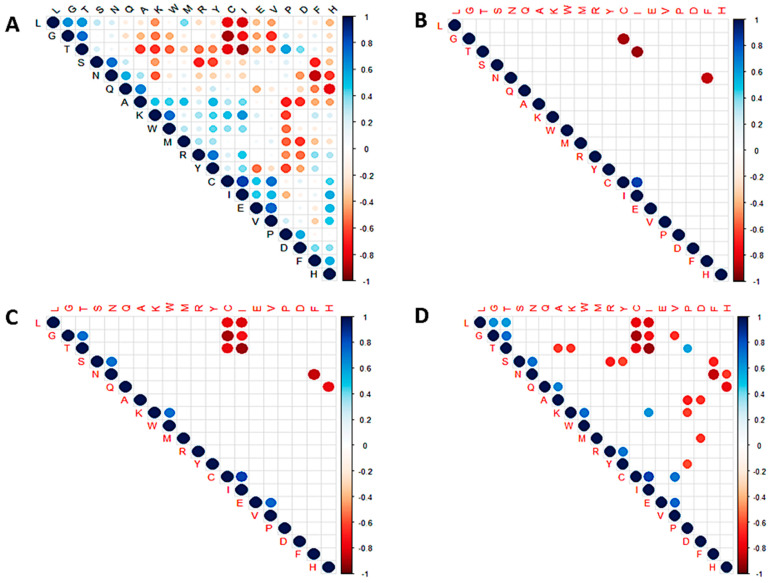
Correlation plots showing the relationship among the 20 standard amino acids of SARS-CoV-2 at (**A**) no *p*-value, (**B**) *p* < 0.001, (**C**) *p* < 0.01, and (**D**) *p* < 0.05.

A correlation plot of the 20 standard amino acids was also generated to investigate the relationship among the residues in all the SARS-CoV-2 proteomes (Figure 4 and Table S3). Correlation coefficients closer to ±1 signify strong correlation [101]. Thus, values greater than 0.7 or less than −0.7 were considered in this study. A previous study that investigated the correlation among amino acids in 204 proteins argued that a correlation coefficient of 0.5 could be considered as strongly correlated since data on structural classes of proteins are a fuzzy set [102]. Herein, the *p*-values were also determined to ascertain whether the correlations are statistically significant. At *p* < 0.001, only four correlation relationships [Cys-Gly, Ile-Thr, Asn-Phe (negative correlations) and Cys-Ile (positive correlation)] were observed (Figure 4B). At *p* < 0.01, a total of 13 relationships existed among the amino acid residues (Figure 4C). Residue pairs Gly-Thr, Ser-Asn, Lys-Trp, Glu-Val, and Cys-Ile were strongly correlated at *p* < 0.01 while Cys-Leu, Cys-Gly, Cys-Thr, Ile-Leu, Ile-Gly, Ile-Thr, Asn-Phe, and His-Gln were negatively correlated (Figure 4C). At *p* < 0.05, a total of 32 correlation relationships were observed among the amino acid residues (Figure 4D).

Threonine had the strongest negative correlation with isoleucine (−0.912) [Figure 4 and Table S3] implying that any mutation that produces a threonine will most likely result in an isoleucine mutating to another amino acid across the proteome of SARS-CoV-2. Although this does not imply that threonine necessarily mutates to isoleucine (or vice versa), several threonine to isoleucine substitutions have been reported in various SARS-CoV-2 proteins [103]. The threonine to isoleucine mutations have been shown in silico to modify the hydrophobicity and structure [103].

In addition, isoleucine had a very strong negative correlation with leucine (−0.818) and glycine (−0.720) while sharing a very strong positive correlation with cysteine (0.835) [Table S3]. Similar trends were observed for cysteine, sharing very strong negative correlations with Leu (−0.779), Thr (−0.784), and Gly (−0.897) and a strong positive correlation with Val (0.704), all at *p* < 0.05 (Table S3). Glycine and threonine were also observed to be strongly correlated with a coefficient of 0.726 (Table S3). Other residue pairs worth mentioning include Val-Glu (0.741), Asn-Ser (0.714), and Thr-Gly (0.726) which had strong correlations while Phe-Asn (−0.853) and His-Gln (−0.78) had strong negative correlations (Table S3).

### 3.7. Test for Normality Using Shapiro and Predicting Probability Using Z-Score

Due to the small variant size (11), the Shapiro-Wilk test was used to determine the normality of the distributions of the various AACs. Normality of the distributions will enable the use of z-scores in predicting the AACs (and possibly frequencies) in future variants or lineages. The skewness and kurtosis of the amino acid residue distributions were also determined (Table 4). All the amino acid residues except methionine had *p*-values greater than 0.05 (Table 4). Met was observed to depart significantly from normality (W = 0.728, *p*-value < 0.05) [Table 4]. However, at *p* < 0.001, Met does not show evidence of non-normality. Met also had the greatest skewness and kurtosis with values of 2.860 and 2.968, respectively. Serine had the most normal distribution (W = 0.973, *p*-value = 0.922) followed by histidine (W = 0.968, *p*-value = 0.865) and glutamine (W = 0.947, *p*-value = 0.610) [Table 4]. Valine (W = 0.922, *p*-value = 0.336) and lysine (W = 0.938, *p*-value = 0.496) had the least skewness of 0.040 and 0.050, respectively (Table 4).

**Figure 5:**
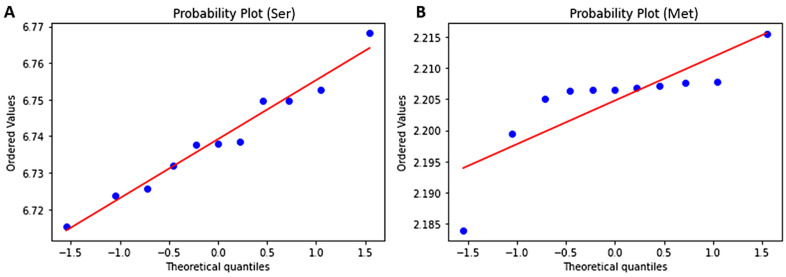
Probability plots to determine the normality for amino acid residues (**A**) Serine and (**B**) Methionine.

Normal probability plots were also generated to analyze how the AAC data deviate from a theoretical distribution (Figure 5 and Figure S3). The probability plot, similar to the quantile-quantile (Q-Q) plot, shows the distribution of the data in comparison to the expected normal distribution. The data is expected to lie along a straight line provided the data is normally distributed. However, if the data is non-normal, a curve is observed, deviating from the straight line. Outliers are the data points that can be seen distant from majority of the points or further away from the straight line. The P-P plot confirmed serine’s normality (Figure 5A) as well as the other amino acids (Figure S3) except methionine. Methionine was observed not to be normally distributed corroborating the results from the Shapiro-Wilk test (Figure 5B). However, with a larger data size, methionine may obey the normality tests.

**Figure 6:**
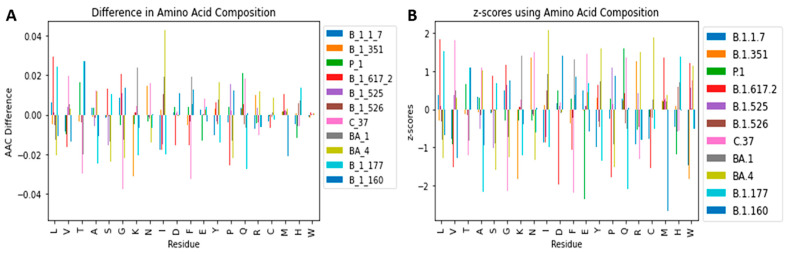
Bar plots showing the (**A**) difference between amino acid composition per variant and the mean amino acid composition and (**B**) z-scores of the amino acid compositions belonging to each of the 11 SARS-CoV-2 variants.

Generally, the normality of the amino acid distribution allows the prediction of AACs of SARS-CoV-2 variants using probability and z-scores. The difference in amino acid composition (variant AAC − mean AAC) (Figure 6A) and standard scores (z-scores) [Figure 6B] were determined with regards to the amino acid residues for the various SARS-CoV-2 variants. The difference between the variant AACs and the average AAC ranged between −0.04 and 0.042 (Figure 6A and Table S4). The highest difference was demonstrated by the isoleucine of variant BA.4, having 0.042% more than the average isoleucine composition. The z-score can be calculated by using Equation (1).

z-score = (variant AAC − mean AAC)/(std of the AAC),(1)

where “std of the AAC” is the standard deviation of a specific amino acid composition (Table S5).

The z-scores of the 20 AACs for all the 11 variants ranged between −2.7 and 2.1 (Figure 6B) representing 0.3 to 98 % chance. The z-score can help predict the most likely combinations of amino acids in emerging variants. For example, from the above equation, there is a 99.29% chance of having a variant with 1.112% Trp since z-score will be 2.448 (~2.45). In addition, there will be a 0.59% chance of having a SARS-CoV-2 variant with 1.108% tryptophan composition since the z-score will be equal to −2.523 (~−2.52). This is not surprising as previous studies have shown that mutating Trp in the SARS-CoV-2 makes the virus highly unstable [87,88]. Moreover, 1.12% is closer to the average AAC (1.11) as compared to 1.08%. The AACs can be beneficial in predicting future viral proteomes which can help in drug discovery, vaccine and protein-protein interaction studies.

### 3.8. Validation of Correlation Analysis

A multiple template alignment of 28 proteome sequences from the 11 variants/lineages used herein were performed and analyzed using webPRANK [66]. The variants which had more than two or three samples in the alignment are labelled as “A”, “B” or “C” in addition to the variant/lineage name to differentiate among them. The lengths of the 28 sequences from the 11 variants/lineages ranged from 14,026 to 14,149 residues while the sequence matrix length was 14,155 columns. The ORF1ab spanned from position 1 to 7096, while ORF1a was from 7097 to 11,501. The spike protein followed to position 12,780 and the ORF3a was from 12,781 to 13,055. The envelope protein’s alignment ended at 13,130, followed by the membrane protein which ended at 13,352, and then ORF6 from 13,353 to 13,413. The alignments of ORF7a and ORF7b ended at positions 13,534 and 13,577, respectively. The ORF8 spanned from 13,578 to 13,698 while the nucleoprotein ended at position 14,117. The last protein on the alignment was ORF10 which ended at position 14,155.

The alignment revealed various deletions across the SARS-CoV-2 proteomes. For the ORF1ab, 82GHV84 (BA.1A), 141KSF143 (BA.4B and B.1.617.2B), 2083S (BA.1B and BA.1C) deletions were observed. The 3675SGF3677 was also deleted in all the samples except for B.1.160, B.1.617.2A, B.1.617.2B, B.1.617.2C, BA.1A, B.1.526A, B.1.177, and B.1.1.7B. Similar deletions were observed in the ORF1a region of the same samples. For the spike region, 24LPP (BA.4A and BA.4B), 69HV (B.1.1.7A, B.1.1.7C, B.1.525A, B.1.525B, B.1.525C, BA.1B, BA.1C, BA.4A, BA.4B, and P.1A), 143VYY (BA.1B and BA.1C), 144Y (P.1A, B.1.1.7A and B.1.1.7C), 145Y (B.1.525A, B and C), 156EF (BA.1A, B.1.617.2A, B.1.617.2B and B.1.617.2C), 217NLVR (BA.1B and BA.1C), 247LLA (B.1.525A, B and C), 252RSYLTPG (C.37A, B and C), and 681QTQTN (B.1.617.2A) deletions were observed. The 211IVREPE was only present in samples BA.1B and BA.1C instead of 217NLVR. 211IVREPE insertion has been previously identified in the BA.1 variant [104]. There was also a 2F deletion in all three samples of the B.1.525 variants in the ORF6 region.

The B.1.617.2C is an ORF8-deleted sample. ORF8-deleted SARS-CoV-2 variants have been shown clinically to have higher transmissibility with milder disease outcomes [105,106,107,108,109]. The ORF8 has been suggested to be involved in pathogenesis and not viral genome replication [110]. Samples B.1.1.7A, B.1.1.7C, and P.1A were also observed to have partial deletions of the ORF8, from 27Q till the end of the ORF8. Deletions affecting the length of the ORF8 is not strange as they have been previously identified [109]. The 119DF120 deletion which has been mentioned in an earlier study [111], was also observed for B.1.617.2A, B.1.617.2B, and BA.1A samples. For the N protein, 3D (B.1.525A, B and C) and 31ERS33 (BA.1B, BA.1C, BA.4A and BA.4B) deletions were observed.

From the alignment of the 28 samples, Trp was observed to be involved in only one mutation (W131L of ORF3a protein) for B.1.351A corroborating the AAC results which suggest that Trp is less likely to mutate (Table 3). Aside W131L, Trp131 has also been reported to mutate to other forms including W131S, W131R, and W131V [112,113]. Herein, all the other 27 samples used for the alignment demonstrated no Trp mutations.

A total of 74 mutations involving Thr or Ile were recorded, with 49 of these being direct Thr-to-Ile or Ile-to-Thr mutations, making up 66.22% of the total instances. Interestingly, there were other indirect mutations involving Thr and Ile. Aside Thr, Ile was observed to only mutate to/from three residues comprising of Met, Val, and Leu. Across the complete proteome, isoleucine was observed to share 5, 4, and 2 mutations with Val, Met, and Leu, respectively. Threonine was also observed to share 6, 4, 3, and 1 mutation(s) with Ala, Asn, Lys, and Arg, respectively. From the earlier correlogram, Thr was predicted to have a negative correlation with Ala and Lys at *p* < 0.05 (Figure 4D), consistent with the alignment results. For the ORF1ab protein, 20 out of 27 (74.07%) mutation instances were Thr-to-Ile or Ile-to-Thr. For the envelope, ORF7a, ORF7b and ORF8 proteins, only one mutation each was observed involving Thr or Ile and they were directly linked. For the N protein, 3 out of 5 mutations (60%) involving Thr or Ile were directly from Thr to Ile.

Cys was observed to mutate to and from Gly in all samples except for BA.1C, BA.4A and BA.4B. In the N protein, G214C (all three C.37 samples) and G215C (BA.1A and B.1.617.2A) mutations were present while C316G (BA.1B) mutation was observed in the ORF1a. R5716C in the ORF1ab (same as R392C in nsp13) [114,115] was observed for BA.4A and BA.4B. In addition, C655Y is observed in both ORF1ab and ORF1a for BA.1C. The results from the alignment show that there is a 66% chance that a Cys will be formed by a mutation from a Gly, as only one out of the three instances Cys was formed by another residue (Arg). A larger data set is required to confirm these findings. However, the same cannot be said for Gly since Gly was observed to also mutate to/from Asp, Ser, Arg, Asn, Val, and Ala. The correlogram also predicted Gln and His to be negatively correlated. From the alignment analysis, Gln and His shared 7 mutations: two in ORF1ab (Q676H and Q5412H), one in ORF1a (Q676H), two in spike (Q683H and Q960H), one in ORF3a (Q57H) and one in the N protein (Q9H). Gln shared 5, 3, 1, and 1 mutation(s) with Arg, Leu, Glu, and Lys, respectively. His was also observed to share 6, 3, and 2 mutations with Tyr, Pro, and Asp, respectively. Although the correlation analysis predicted a strong negative correlation between Asn and Phe (Figure 4), there were no direct mutations between the two residues. They could probably be indirectly related.

### 3.9. Limitations of the Study

Similar to all other research, this study also has its shortcomings and limitations. The relatively small sample size used herein is a major shortcoming of this study since they may not accurately reflect the overall frequency of each amino acid in the proteomes although the use of AACs could help mitigate this challenge. Furthermore, the limited number of variants used (11) does not provide complete analysis of all SARS-CoV-2 variants. The uneven sample sizes of the variants could also influence the overall accuracy of the results reported herein. Nonetheless, this study reports correlations among the amino acid residues that can be further investigated on a larger sample size to corroborate these findings. Robust and more accurate strategies/methods for validating the correlations must be developed in order to identify or measure indirectly linked residues.

Furthermore, the study did not investigate the correlation existing among the amino acids taking epistasis into consideration. A mutation in one protein can influence another mutation in a different protein of the same organism since proteins often interact with each other to perform specific functions, and changes in one protein can impact the behavior or function of others. Efforts are ongoing to elucidate epistasis in the SARS-CoV-2 genome as this phenomenon is still in its nascent stages for this virus [116,117]. A recent study showed that several pairwise epistases exist among eight viral genes [116]. The impact of one mutation on another can be complex and may depend on a variety of factors, including the specific proteins involved, the context of the mutations, and the environment. Understanding these interactions is important for understanding the evolution of viruses and for developing effective strategies to control the spread of infectious diseases. This molecular mechanism also contributes to the evolution of SARS-CoV-2 and the emergence of new viral variants [116]. Understanding these SARS-CoV-2 mutation mechanisms is important for developing effective strategies to control the spread of COVID-19. Future studies could evaluate the correlations existing among the amino acid residues taking into consideration epistatically linked pairs.

### 3.10. Potential Implications of the Study and Future Perspective

This study showed that relationships exist among the amino acid residues in the SARS-CoV-2 proteome. These relationships in addition to knowledge from molecular mechanisms on viral mutations and environmental factors, can help in designing lab-made mutants for mutational studies. Previous studies have successfully predicted structural classes of proteins using AAC-based models [14,15]. Although predicting viral mutations are complex due to the many factors involved, optimizing machine-learning based models which use up-to-date resources can improve the success rate, which will in turn help in drug and vaccine research and design. The AACs along with evolutionary information can be exploited to predict protein secondary structures [21], and later for protein synthesis, study protein-protein and protein-drug interactions [19,20]. For diagnostics purposes, the AACs can help classify strains or variants since each variant has specific AACs for the amino acid residues.

## 4. Conclusions

This study showed that correlation relationships exist among the 20 standard amino acids in the SARS-CoV-2 proteome. Herein, the frequencies of the amino acid residues in 1673 complete samples belonging to 11 SARS-CoV-2 variants were carefully computed and analyzed. The AACs revealed that leucine and tryptophan were the most and least abundant, respectively. Correlation analysis also showed that cysteine and isoleucine have very strong negative correlations with leucine, glycine, and threonine at *p* < 0.01. Threonine and isoleucine were observed to have the strongest negative correlation of −0.912 at *p* < 0.001. At *p* < 0.001, isoleucine and cysteine had a very strong positive correlation of 0.835. Special attention must be given to these amino acids when predicting mutations in the SARS-CoV-2. In addition, we show that the AAC is able to predict the ancestry and relationships among SARS-CoV-2 variants. The AAC analysis and correlations reported herein will aid in predicting and classifying viral strains. The AACs of the variants were also observed to be normally distributed which can be leveraged to predict future proteome contents using probability and z-scores.

## Figures and Tables

**Table 1:** Details of proteome samples of SARS-CoV-2 variants obtained from NCBI.

Nextstrain Clade	Pango Lineage	WHO Label	Number of Files		Remarks
			Before Cleaning	After Cleaning	
20I (Alpha, V1)	B.1.1.7	Alpha	830	706	-
20H (Beta, V2)	B.1.351	Beta	16	4	-
20J (Gamma, V3)	P.1	Gamma	79	73	-
21A (Delta)	B.1.617.2	Delta	100	43	-
21B (Kappa)	B.1.617.1	Kappa	1	-	Part of B.1.617.2
21C (Epsilon)	B.1.427, B.1.429	Epsilon	0	-	-
21D (Eta)	B.1.525	Eta	13	13	-
21F (Iota)	B.1.526	Iota	3085	773	-
21G (Lambda)	C.37	Lambda	19	8	-
21H (Mu)	B.1.621	Mu	0	-	-
21K (Omicron)	BA.1	Omicron	26	13	-
21L (Omicron)	BA.2	Omicron	23	-	Part of BA.1
22A (Omicron)	BA.4	Omicron	3	2	-
22B (Omicron)	BA.5	Omicron	23	0	-
22C (Omicron)	BA.2.12.1	Omicron	0	-	-
22D (Omicron)	BA.2.75	Omicron	0	-	-
20E (EU1)	B.1.177		1	1	-
20B/S:732A	B.1.1.519		0	-	-
20A/S:126A	B.1.620		0	-	-
20A.EU2	B.1.160		1	1	-
20A/S:439K	B.1.258		0	-	-
20A/S:98F	B.1.221		0	-	-
20C/S:80Y	B.1.367		0	-	-
20B/S:626S	B.1.1.277		0	-	-
20B/S:1122L	B.1.1.302		0	-	-

**Table 2:** Average amino acid count for each of the 11 lineages used in this study. The values presented here are rounded up to 3 decimal places.

Residues	SARS-CoV-2 Variants										
	B.1.1.7	B.1.351	P.1	B.1.617.2	B.1.525	B.1.526	C.37	BA.1	BA.4	B.1.177	B.1.160
M	311.508	312	311.973	312.512	312	310.979	312	311.923	312	312	309
E	679.016	680	678.123	677.721	680	680.049	681	680.385	680	681	680
S	950.745	952.75	951.849	952.535	950.615	952.779	956.75	950.846	949	955	955
L	1363.72	1365	1366.75	1366.58	1365.69	1364.88	1363.12	1363.12	1362	1370	1365
V	1148.64	1153	1150.74	1147.14	1152.62	1152.57	1154.62	1152.77	1152	1154	1151
P	553.214	556	555.438	549.953	557	556.211	555	552.923	551.5	557	557
G	838.057	840	837.781	839.465	840	839.507	833	836.615	835	840	841
F	707.078	707	708.301	703.884	707	707.248	703	710.385	709.5	709	710
N	763.834	767.5	764.932	765.395	765.154	765.133	767.5	763	763	765	766
K	836.659	834	837.63	836.535	838.846	838.477	838.75	841.615	842	836	838
T	1060.65	1059	1061.75	1056.91	1061	1058.85	1055	1056.46	1052	1064	1064
H	263.683	265	263.192	262.047	264	265.706	264	265.846	267	267	265
Q	514.037	515	517.534	514.14	514	513.877	517	513.538	513	511	515
R	479.565	483	480.959	480.837	482	481.034	480	482.923	483	481	481
D	718.683	718.5	720.493	716.093	720	719.699	720	719.769	719.5	722	722
A	964.416	965.75	966.329	965.698	964.923	965.577	967.375	965.462	967	963	965
C	432.669	434	433.904	432.047	434	433.875	435	434.154	435	434	434
Y	640.779	644	645.589	642.93	643	642.849	642	644.538	645.5	642	643
I	727.377	731.75	728.986	727.116	729.154	732.816	733.875	734	737	729	731
W	156.469	156.75	156.959	156.721	157	156.951	157	157	157	157	157

**Table 3:** Average amino acid composition for each SARS-CoV-2 variant. Values are presented up to 3 decimal places.

Residues	Average Amino Acid Composition (AAC) of SARS-CoV-2 Variants											Avg AAC
	B.1.1.7	B.1.351	P.1	B.1.617.2	B.1.525	B.1.526	C.37	BA.1	BA.4	B.1.177	B.1.160	
M	2.208	2.207	2.206	2.215	2.207	2.199	2.207	2.206	2.208	2.205	2.184	2.205
E	4.812	4.810	4.796	4.804	4.810	4.810	4.817	4.813	4.812	4.813	4.806	4.809
S	6.738	6.738	6.732	6.753	6.724	6.739	6.768	6.726	6.715	6.750	6.750	6.739
L	9.664	9.653	9.666	9.688	9.660	9.653	9.643	9.647	9.638	9.683	9.647	9.658
V	8.140	8.154	8.139	8.132	8.153	8.152	8.168	8.154	8.152	8.156	8.135	8.149
P	3.921	3.932	3.928	3.899	3.940	3.934	3.926	3.911	3.902	3.937	3.937	3.924
G	5.939	5.941	5.925	5.951	5.941	5.938	5.893	5.918	5.909	5.937	5.944	5.930
F	5.011	5	5.009	4.990	5.001	5.002	4.973	5.025	5.021	5.011	5.018	5.005
N	5.413	5.428	5.410	5.426	5.412	5.411	5.429	5.397	5.310	5.407	5.414	5.413
K	5.929	5.898	5.924	5.930	5.933	5.930	5.933	5.953	5.958	5.909	5.923	5.929
T	7.517	7.489	7.509	7.492	7.505	7.489	7.463	7.473	7.444	7.520	7.520	7.493
H	1.869	1.874	1.861	1.858	1.867	1.879	1.868	1.880	1.889	1.887	1.873	1.873
Q	3.643	3.642	3.660	3.645	3.636	3.634	3.657	3.632	3.630	3.612	3.640	3.639
R	3.399	3.416	3.402	3.409	3.409	3.402	3.396	3.416	3.418	3.400	3.400	3.406
D	5.093	5.081	5.096	5.076	5.093	5.090	5.093	5.091	5.091	5.103	5.103	5.092
A	6.834	6.830	6.834	6.846	6.825	6.829	6.843	6.829	6.843	6.806	6.820	6.831
C	3.066	3.069	3.069	3.063	3.070	3.069	3.077	3.071	3.078	3.067	3.067	3.070
Y	4.541	4.554	4.566	4.558	4.548	4.547	4.542	4.559	4.568	4.537	4.544	4.551
I	5.155	5.175	5.156	5.155	5.157	5.183	5.192	5.192	5.215	5.152	5.166	5.173
W	1.109	1.109	1.110	1.111	1.110	1.110	1.111	1.110	1.111	1.110	1.110	1.110

**Table 4:** Test for normality for the amino acid compositions using Shapiro-Wilk test, skewness and kurtosis. The values presented are rounded to 3 decimal places.

Residues	Skewness		Kurtosis	Shapiro Wilk Test	
	Statistic	*p*-Value		Test Statistic (W)	*p*-Value
M	2.860	0.004	2.968	0.728	0.001
E	−1.834	0.067	0.978	0.906	0.217
S	0.495	0.621	−0.510	0.973	0.922
L	1.153	0.249	−0.661	0.931	0.425
V	0.040	0.968	−0.772	0.922	0.336
P	−1.301	0.193	−0.896	0.884	0.115
G	−1.747	0.081	−0.095	0.879	0.102
F	−1.463	0.143	0.167	0.936	0.473
N	0.251	0.802	−0.936	0.923	0.348
K	0.050	0.960	−0.203	0.938	0.496
T	−1.128	0.259	−0.643	0.923	0.341
H	0.278	0.781	−0.961	0.968	0.865
Q	−0.562	0.574	0.210	0.947	0.610
R	0.631	0.528	−1.401	0.892	0.147
D	−0.877	0.380	−0.116	0.911	0.250
A	−1.226	0.220	0.102	0.938	0.493
C	1.338	0.181	−0.087	0.891	0.143
Y	0.568	0.570	−1.220	0.937	0.481
I	1.383	0.167	−0.520	0.873	0.085
W	−1.054	0.292	−0.759	0.927	0.384

## Data Availability

Not applicable.

## Supplementary Materials

The following supporting information can be downloaded at: https://www.mdpi.com/article/10.3390/biomedicines11020512/s1, Figure S1: Bar plots showing the average frequencies of the amino acid residues belonging to SARS-CoV-2 variants (A) B.1.1.7, (B) B.1.351, (C) P.1, (D) B.1.617.2, (E) B.1.525, (F) B.1.526, (G) C.37, (H) BA.1, (I) BA.4, (J) B.1.177, and (K) B.1.160; Figure S2: Phylogenetic relationships among SARS-CoV-2 clades as determined by Nextstrain; Figure S3: Probability plots to determine the normality for amino acid residues (A) Alanine, (B) Cysteine, (C) Aspartic acid, (D) Glutamic acid, (E) Phenylalanine, (F) Glycine, (G) Histidine, (H) Isoleucine, (I) Lysine, (J) Leucine, (K) Asparagine, (L) Proline, (M) Glutamine, (N) Arginine, (O) Threonine, (P) Valine, (Q) Tryptophan, and (R) Tyrosine; Table S1: Average and standard deviation of each amino acid for all the SARS-CoV-2 variants used in this study; Table S2: Correlation among the SARS-CoV-2 variants using their amino acid compositions; Table S3: Correlation matrix among the 20 standard amino acids using their compositions; Table S4: Calculated z-scores of the amino acid residues belonging to the 11 SARS-CoV-2 variants using their compositions. Z-score values are presented in 3 decimal places; Table S5: Average and standard deviation of the amino acid compositions.
