# Supplementary material for: Computational Analysis Predicts Correlations among Amino Acids in SARS-CoV-2 Proteomes
Source: Biomedicines. 2023 Feb 10;11(2):512. doi: 10.3390/biomedicines11020512 (PMC9953644; doi:10.3390/biomedicines11020512)
Supplement: Supplementary file 1 [file biomedicines-11-00512-s001.zip › biomedicines-2193445-supplementary.pdf]

Supplementary Materials

Supplementary Tables

Table S1: Average and standard deviation of each amino acid for all the SARS-CoV-2 variants used in this study.

Residue	Average	Standard Deviation		Residue	Average	Standard Deviation
**M**	311.627	0.950		**T**	1059.056	3.743
**E**	679.754	1.057		**H**	264.770	1.561
**S**	952.534	2.296		**Q**	514.375	1.794
**L**	1365.131	2.162		**R**	481.393	1.187
**V**	1151.736	2.235		**D**	719.703	1.641
**P**	554.658	2.421		**A**	965.503	1.207
**G**	838.220	2.467		**C**	433.877	0.864
**F**	707.491	2.357		**Y**	643.290	1.495
**N**	765.132	1.512		**I**	731.098	3.110
**K**	838.047	2.337		**W**	156.895	0.175

Table S2: Correlation among the SARS-CoV-2 variants using their amino acid compositions.

	**B.1.1.7**	**B.1.351**	**P.1**	**B.1.617.2**	**B.1.525**	**B.1.526**	**C.37**	**BA.1**	**BA.4**	**B.1.177**	**B.1.160**	**mean**
**B.1.1.7**	1	0.999981422	0.999991054	0.999979808	0.999992855	0.999987206	0.999940845	0.999967045	0.99992745	0.999979207	0.999989344	0.999993093
**B.1.351**	0.999981422	1	0.999979229	0.999973658	0.999985361	0.999989661	0.99995597	0.999962989	0.999934604	0.999970094	0.999977168	0.999990713
**P.1**	0.999991054	0.999979229	1	0.999976444	0.999987597	0.999980531	0.99994231	0.999964217	0.999928386	0.999964656	0.999981373	0.999989413
**B.1.617.2**	0.999979808	0.999973658	0.999976444	1	0.999971597	0.999969759	0.999937989	0.999950648	0.999921544	0.999948	0.999956787	0.999979456
**B.1.525**	0.999992855	0.999985361	0.999987597	0.999971597	1	0.999991599	0.999942895	0.999971173	0.999934668	0.999979355	0.999984728	0.999993596
**B.1.526**	0.999987206	0.999989661	0.999980531	0.999969759	0.999991599	1	0.999964139	0.999983139	0.999958809	0.999974377	0.999985413	0.999997475
**C.37**	0.999940845	0.99995597	0.99994231	0.999937989	0.999942895	0.999964139	1	0.99995049	0.99994118	0.999914871	0.999931678	0.999964526
**BA.1**	0.999967045	0.999962989	0.999964217	0.999950648	0.999971173	0.999983139	0.99995049	1	0.999989204	0.999944086	0.999958092	0.999984403
**BA.4**	0.99992745	0.999934604	0.999928386	0.999921544	0.999934668	0.999958809	0.99994118	0.999989204	1	0.99989574	0.999915321	0.999957638
**B.1.177**	0.999979207	0.999970094	0.999964656	0.999948	0.999979355	0.999974377	0.999914871	0.999944086	0.99989574	1	0.999978336	0.999976054

**B.1.160**	0.999989344	0.999977168	0.999981373	0.999956787	0.999984728	0.999985413	0.999931678	0.999958092	0.999915321	0.999978336	1	0.999986002
**mean**	0.999993093	0.999990713	0.999989413	0.999979456	0.999993596	0.999997475	0.999964526	0.999984403	0.999957638	0.999976054	0.999986002	1

Table S3: Correlation matrix among the 20 standard amino acids using their compositions.

Residue	M	E	S	L	V	P	G	F	N	K
**M**	1									
**E**	0.076354	1								
**S**	-0.11991	0.186406	1							
**L**	0.388378	-0.36135	0.278265	1						
**V**	0.16442	0.740921	0.089172	-0.43586	1					
**P**	-0.55268	-0.00715	0.183451	-0.02407	0.240467	1				
**G**	-0.15657	-0.43265	-0.02719	0.622425	-0.65711	0.222757	1			
**F**	-0.35239	-0.18157	-0.68778	-0.17658	-0.31543	-0.08257	0.161648	1		
**N**	0.140411	-0.00973	0.714094	0.213816	0.020429	0.145729	0.14648	-0.85325	1	
**K**	0.160416	0.206744	-0.4475	-0.46002	0.060203	-0.61399	-0.51277	0.266515	-0.56161	1
**T**	-0.34756	-0.40423	0.217895	0.605728	-0.50508	0.579677	0.726114	0.137014	0.098713	-0.63719
**H**	-0.23448	0.512731	-0.35322	-0.38615	0.460621	0.036228	-0.26029	0.545091	-0.62424	0.190483
**Q**	0.106424	-0.40675	0.263608	-0.15248	-0.19129	-0.08412	-0.24485	-0.4925	0.540306	0.0025
**R**	0.326407	-0.01194	-0.69391	-0.25079	0.052847	-0.47164	0.001813	0.36651	-0.30521	0.321041
**D**	-0.63071	0.099716	0.052441	-0.16773	0.117416	0.551937	-0.18456	0.387087	-0.4502	-0.02556
**A**	0.498091	-0.07142	0.000643	-0.21684	-0.09607	-0.69861	-0.38045	-0.41004	0.321319	0.476618
**C**	0.037906	0.47972	-0.18769	-0.77921	0.703698	-0.10822	-0.89753	-0.07032	-0.18647	0.490854
**Y**	0.326262	-0.52118	-0.60757	-0.18023	-0.25526	-0.59111	-0.18757	0.303303	-0.29974	0.433974
**I**	-0.01835	0.453188	-0.28035	-0.81809	0.510621	-0.38341	-0.72045	0.114201	-0.27754	0.616241
**W**	0.298096	0.006879	-0.08413	-0.07783	0.076135	-0.52128	-0.40764	-0.16601	-0.21032	0.72863

Residue	T	H	Q	R	D	A	C	Y	I	W
**T**	1									
**H**	-0.31718	1								
**Q**	-0.08767	-0.77984	1							
**R**	-0.53121	0.303377	-0.24561	1						
**D**	0.38474	0.368365	-0.27377	-0.53767	1					
**A**	-0.62722	-0.43747	0.663367	0.212429	-0.64584	1				
**C**	-0.78447	0.434997	0.082236	0.207102	0.167117	0.30318	1			
**Y**	-0.49369	-0.04787	0.257668	0.695842	-0.42306	0.513429	0.222824	1		
**I**	-0.91247	0.555705	-0.0463	0.46132	-0.10256	0.397203	0.835165	0.381116	1	
**W**	-0.55959	-0.06489	0.065332	0.192837	-0.14961	0.447948	0.3517	0.413138	0.377135	1

Table S4: Calculated z-scores of the amino acid residues belonging to the 11 SARS-CoV-2 variants using their compositions. Z-score values are presented in 3 decimal places.

Residue	B.1.1.7	B.1.351	P.1	B.1.617.2	B.1.525	B.1.526	C.37	BA.1	BA.4	B.1.177	B.1.160
L	0.380	-0.303	0.507	1.843	0.089	-0.315	-0.961	-0.789	-1.289	1.524	-0.687
V	-0.780	0.526	-0.920	-1.525	0.380	0.292	1.810	0.490	0.297	0.701	-1.272
T	0.959	-0.138	0.665	-0.014	0.476	-0.162	-1.195	-0.817	-1.967	1.096	1.096
A	0.320	-0.089	0.302	1.306	-0.515	-0.156	1.084	-0.176	1.021	-2.165	-0.931
S	-0.096	-0.079	-0.475	0.886	-1.014	-0.036	1.918	-0.901	-1.581	0.689	0.689
G	0.496	0.580	-0.295	1.173	0.628	0.404	-2.144	-0.732	-1.243	0.365	0.767
K	0.002	-1.824	-0.295	0.062	0.241	0.061	0.250	1.396	1.701	-1.213	-0.382
N	-0.017	1.351	-0.307	1.171	-0.118	-0.169	1.493	-1.525	-1.318	-0.609	0.047
I	-0.861	0.123	-0.812	-0.870	-0.732	0.505	0.923	0.933	2.066	-0.980	-0.294
D	0.160	-1.349	0.487	-1.975	0.098	-0.223	0.190	-0.104	-0.078	1.397	1.397
F	0.366	-0.371	0.270	-1.057	-0.323	-0.230	-2.190	1.300	1.017	0.370	0.848
E	0.494	-0.036	-2.350	-0.864	0.085	0.083	1.464	0.578	0.448	0.677	-0.580
Y	-0.991	0.309	1.423	0.630	-0.315	-0.452	-0.938	0.746	1.589	-1.343	-0.658
P	-0.258	0.554	0.291	-1.787	1.087	0.676	0.137	-0.927	-1.518	0.873	0.873
Q	0.276	0.222	1.590	0.419	-0.273	-0.359	1.368	-0.516	-0.690	-2.086	0.047
R	-0.917	1.258	-0.536	0.356	0.428	-0.464	-1.292	1.255	1.501	-0.795	-0.795
C	-0.768	-0.082	-0.195	-1.530	0.014	-0.234	1.685	0.264	1.879	-0.516	-0.516
M	0.362	0.223	0.214	1.361	0.263	-0.681	0.303	0.196	0.383	0.044	-2.666
H	-0.457	0.087	-1.177	-1.552	-0.591	0.597	-0.565	0.711	1.602	1.376	-0.032
W	-1.460	-1.835	0.077	1.204	0.557	0.021	0.752	0.572	1.143	-0.516	-0.516

Table S5: Average and standard deviation of the amino acid compositions.

Residue	Average AAC (%)	Standard Deviation		Residue	Average AAC (%)	Standard Deviation
**M**	2.20476	0.00782399		**T**	7.49281	0.0247689
**E**	4.80925	0.00562207		**H**	1.87324	0.0100407
**S**	6.73917	0.0151228		**Q**	3.6392	0.013251
**L**	9.6583	0.0159854		**R**	3.40585	0.00794262
**V**	8.14852	0.0107471		**D**	5.09189	0.0078317
**P**	3.92419	0.0142934		**A**	6.83093	0.0114516
**G**	5.93041	0.0175641		**C**	3.06968	0.00449508
**F**	5.00549	0.0147773		**Y**	4.55127	0.0103095
**N**	5.4133	0.0107792		**I**	5.17251	0.0206201
**K**	5.92918	0.0170094		**W**	1.11003	0.0008046

Supplementary Figures

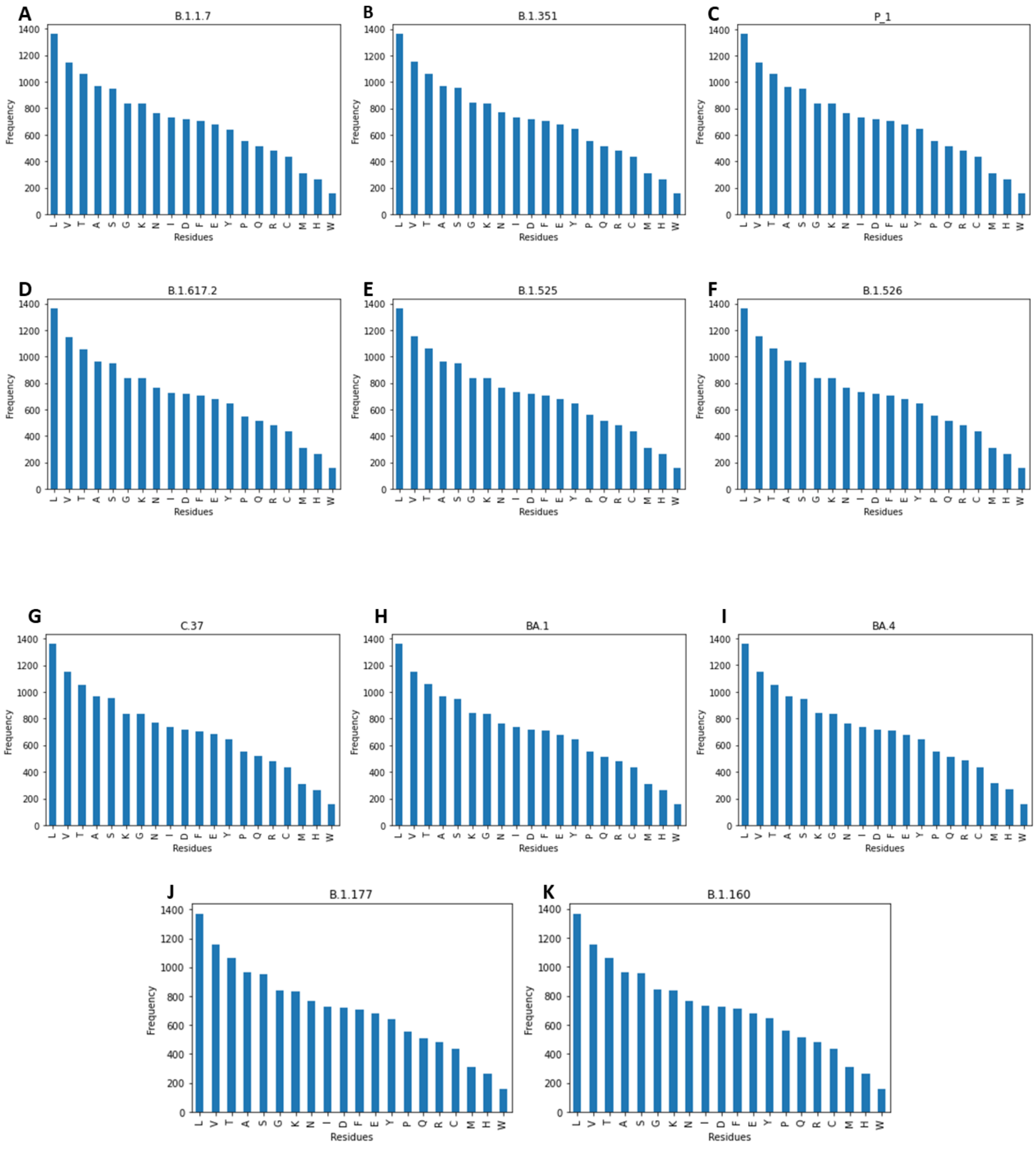

Figure S1: Bar plots showing the average frequencies of the amino acid residues belonging to SARS-CoV-2 variants (A) B.1.1.7, (B) B.1.351, (C) P.1, (D) B.1.617.2, (E) B.1.525, (F) B.1.526, (G) C.37, (H) BA.1, (I) BA.4, (J) B.1.177, and (K) B.1.160.

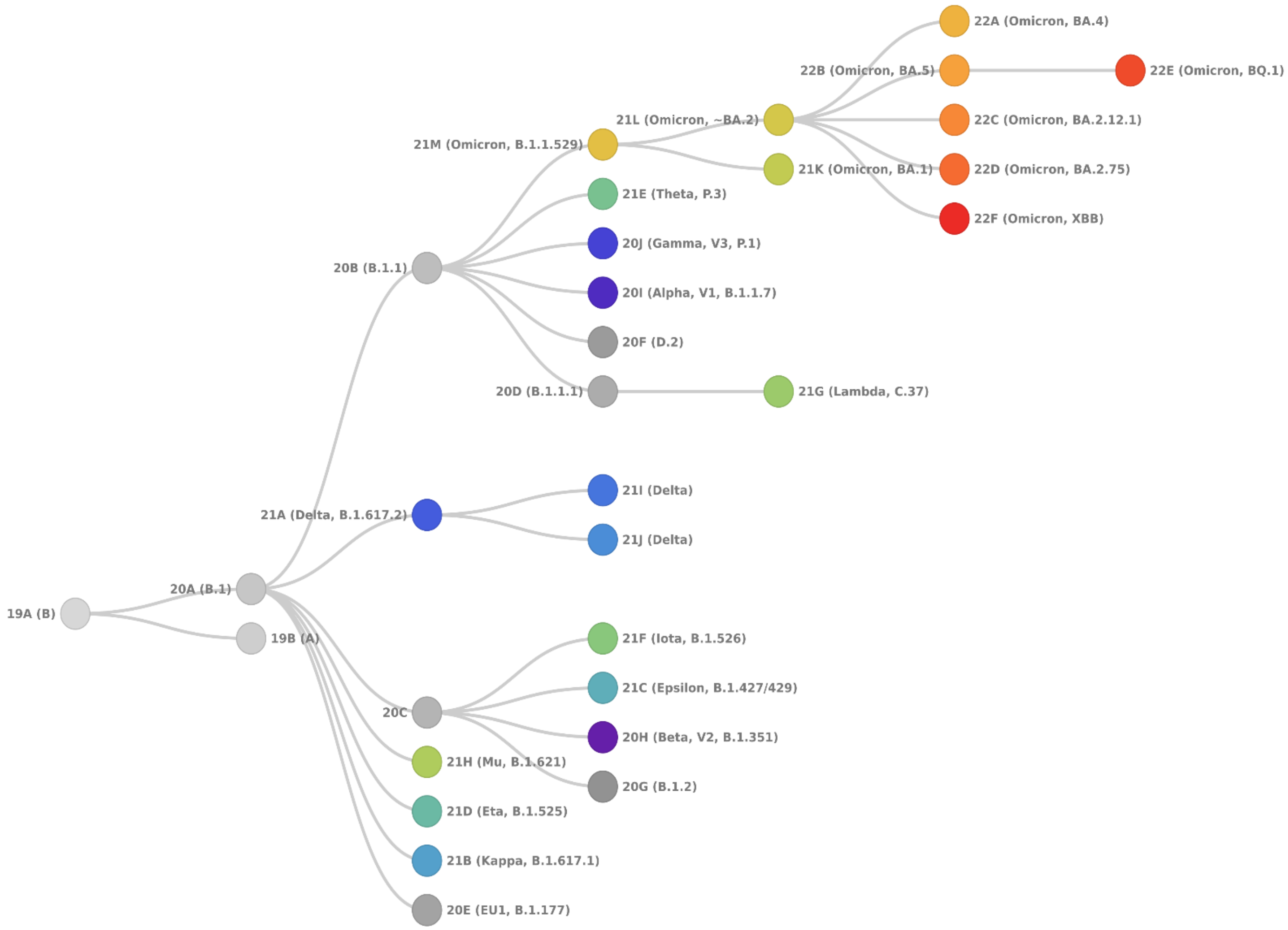

Figure S2: Phylogenetic relationships among SARS-CoV-2 clades as determined by Nextstrain (https://github.com/nextstrain/ncov-clades-schema) [1].

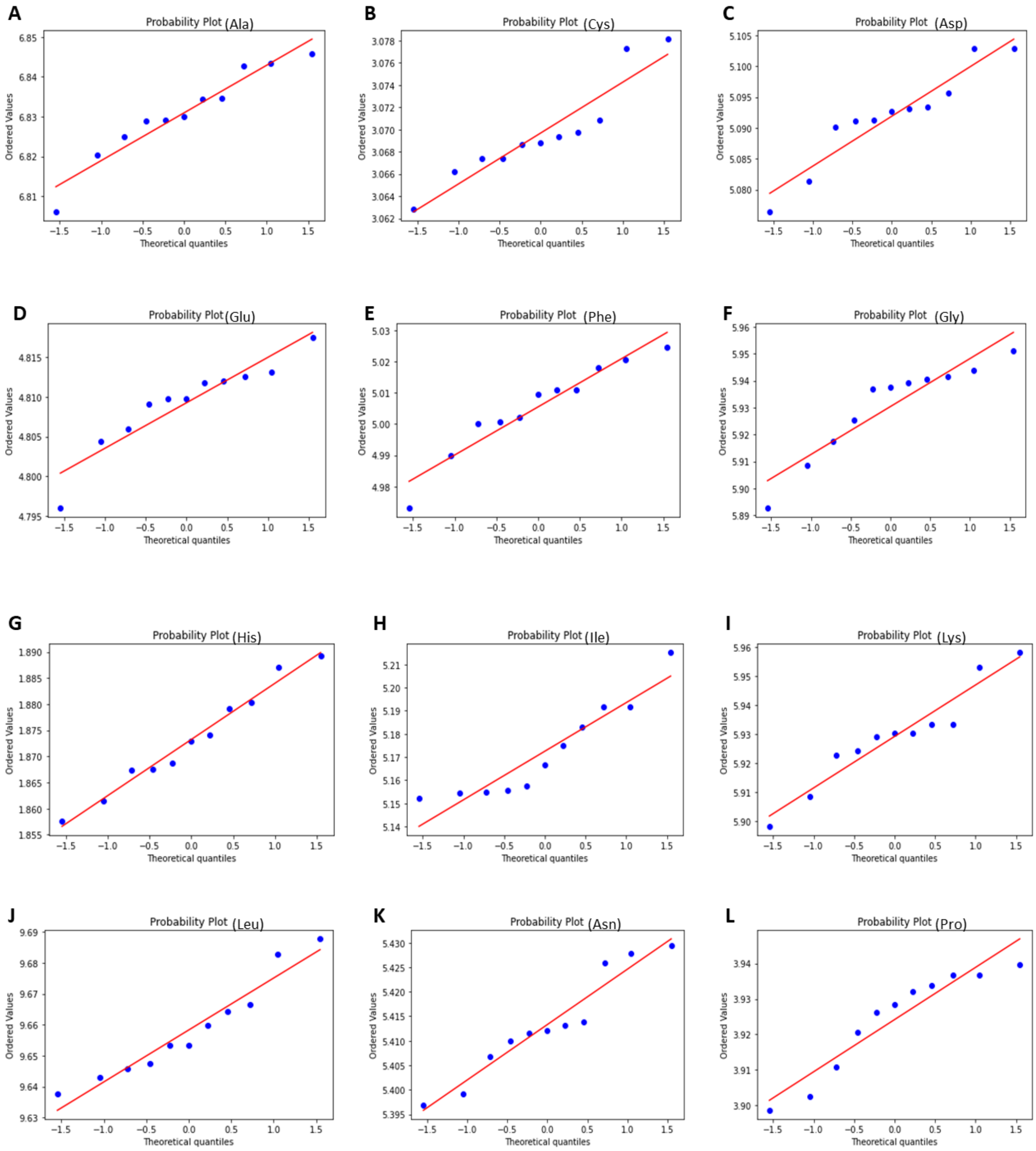
A
Probability Plot (Ala)
B
Probability Plot (Cys)
C
Probability Plot (Asp)
D
Probability Plot (Glu)
E
Probability Plot (Phe)
F
Probability Plot (Gly)
G
Probability Plot (His)
H
Probability Plot (Ile)
I
Probability Plot (Lys)
J
Probability Plot (Leu)
K
Probability Plot (Asn)
L
Probability Plot (Pro)
Ordered Values
Theoretical quantiles

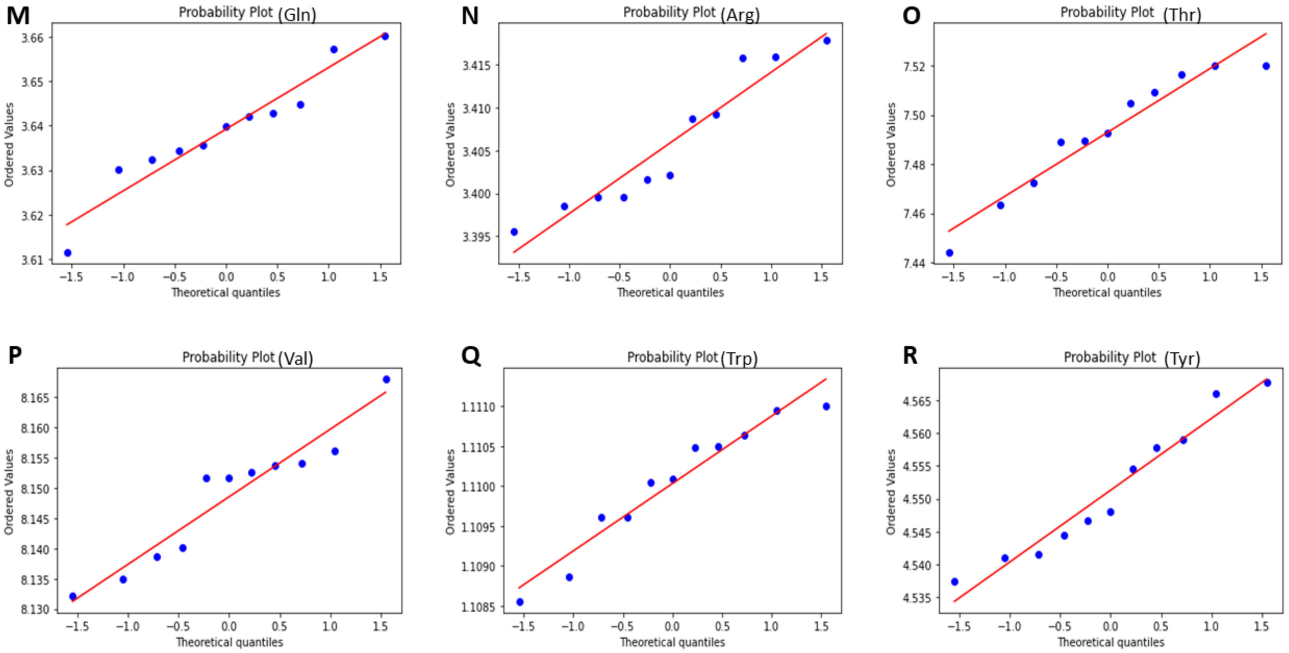

Figure S3: Probability plots to determine the normality for amino acid residues (A) Alanine, (B) Cysteine, (C) Aspartic acid, (D) Glutamic acid, (E) Phenylalanine, (F) Glycine, (G) Histidine, (H) Isoleucine, (I) Lysine, (J) Leucine, (K) Asparagine, (L) Proline, (M) Glutamine, (N) Arginine, (O) Threonine, (P) Valine, (Q) Tryptophan, and (R) Tyrosine.

References

1. Hadfield, J.; Megill, C.; Bell, S.M.; Huddleston, J.; Potter, B.; Callender, C.; Sagulenko, P.; Bedford, T.; Neher, R.A. Nextstrain: real-time tracking of pathogen evolution. *Bioinformatics* **2018**, *34*, 4121–4123, doi:10.1093/bioinformatics/bty407.
